# Supplementary material for: Neuronal genes deregulated in Cornelia de Lange Syndrome respond to removal and re-expression of cohesin
Source: Nat Commun. 2021 May 18;12:2919. doi: 10.1038/s41467-021-23141-9 (PMC8131595; doi:10.1038/s41467-021-23141-9)
Supplement: Supplementary file 1 — Supplementary Information [file 41467_2021_23141_MOESM1_ESM.pdf]

Supplementary information

Neuronal genes deregulated in Cornelia de Lange Syndrome respond to removal and re-expression of cohesin

Felix D. Weiss[1,$], Lesly Calderon[1,$], Yi-Fang Wang[2], Radina Georgieva[1,3], Ya Guo[1], Nevena Cvetesic[3], Maninder Kaur[4], Gopuraja Dharmalingam[2], Ian D. Krantz[4,5,6], Boris Lenhard[3,7], Amanda G. Fisher[1], Matthias Merkenschlager[1] *

[1] Lymphocyte Development Group, Epigenetics Section, MRC London Institute of Medical Sciences, Institute of Clinical Sciences, Faculty of Medicine, Imperial College London, London, UK.

[2] MRC London Institute of Medical Sciences, Institute of Clinical Sciences, Faculty of Medicine, Imperial College London, London, UK.

[3] Computational Regulatory Genomics Group, Integrative Biology Section, MRC London Institute of Medical Sciences, Institute of Clinical Sciences, Faculty of Medicine, Imperial College London, London, UK.

[4] Division of Human Genetics, The Department of Pediatrics, The Children's Hospital of Philadelphia, Philadelphia, Pennsylvania, 19104, USA.

[5] The Perelman School of Medicine at The University of Pennsylvania, Philadelphia, Pennsylvania, 19104, USA.

[6] Department of Pathology and Laboratory Medicine, The Children's Hospital of Philadelphia, Philadelphia, Pennsylvania, 19104, USA.

[7] Sars International Centre for Marine Molecular Biology, University of Bergen, N-5008 Bergen, Norway

[$] These authors contributed equally

* email: matthias.merkenschlager@lms.mrc.ac.uk

Supplementary Figures and Legends

a

ID	Origin	Condition	Age	Gender	Mutation
5345	NIH	control	48	Female	
4788	NIH	control	48	Female	
1739	NIH	control	48	Female	
2082	NIH	CdLS	48	Female	NIPBL 7669T>C ; C2557R
SD047/15	Edinburgh	control	19	Male	
SD023/08	Edinburgh	control	24	Female	
SD030/11	Edinburgh	control	30	Male	
CDL-380P	CHOP	CdLS	19	Male	NIPBL 2965_2966insA
CDL-744P	CHOP	CdLS	24	Female	NIPBL 5961delT
CDL-764P	CHOP	CdLS	31	Female	none detected

b

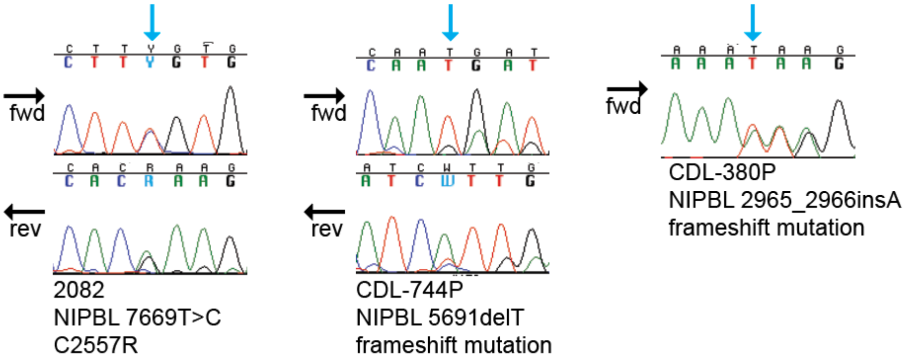

c

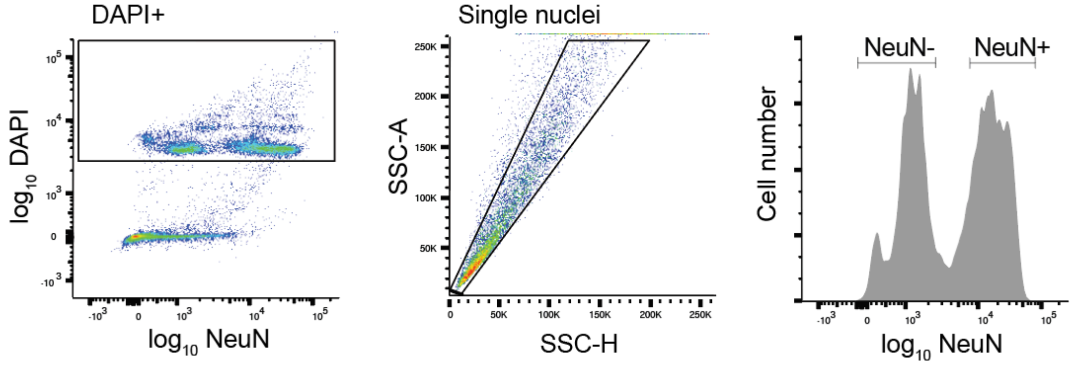

Supplementary Figure 1. Characterization of CdLS patients and isolation of nuclei

a) Metadata for patient samples, including ID, origin, disease status, patient age, patient gender and mutational status if applicable.

b) Sanger sequencing of CdLS patient samples 2082, CDL-744P and CDL-380P confirming mutations initially identified by RNAseq. Black arrows indicate direction of Sanger sequencing reaction. Blue arrow indicates site of mutation.

c) Flow cytometric parameters used to purify neuronal nuclei from whole frozen post-mortem tissue. DDAPI-positive nuclei were gated for single nuclei (70-90% of nuclei) and sorted into NeuN-positive (30-50% of nuclei) and NeuN-negative (50-70% of nuclei).

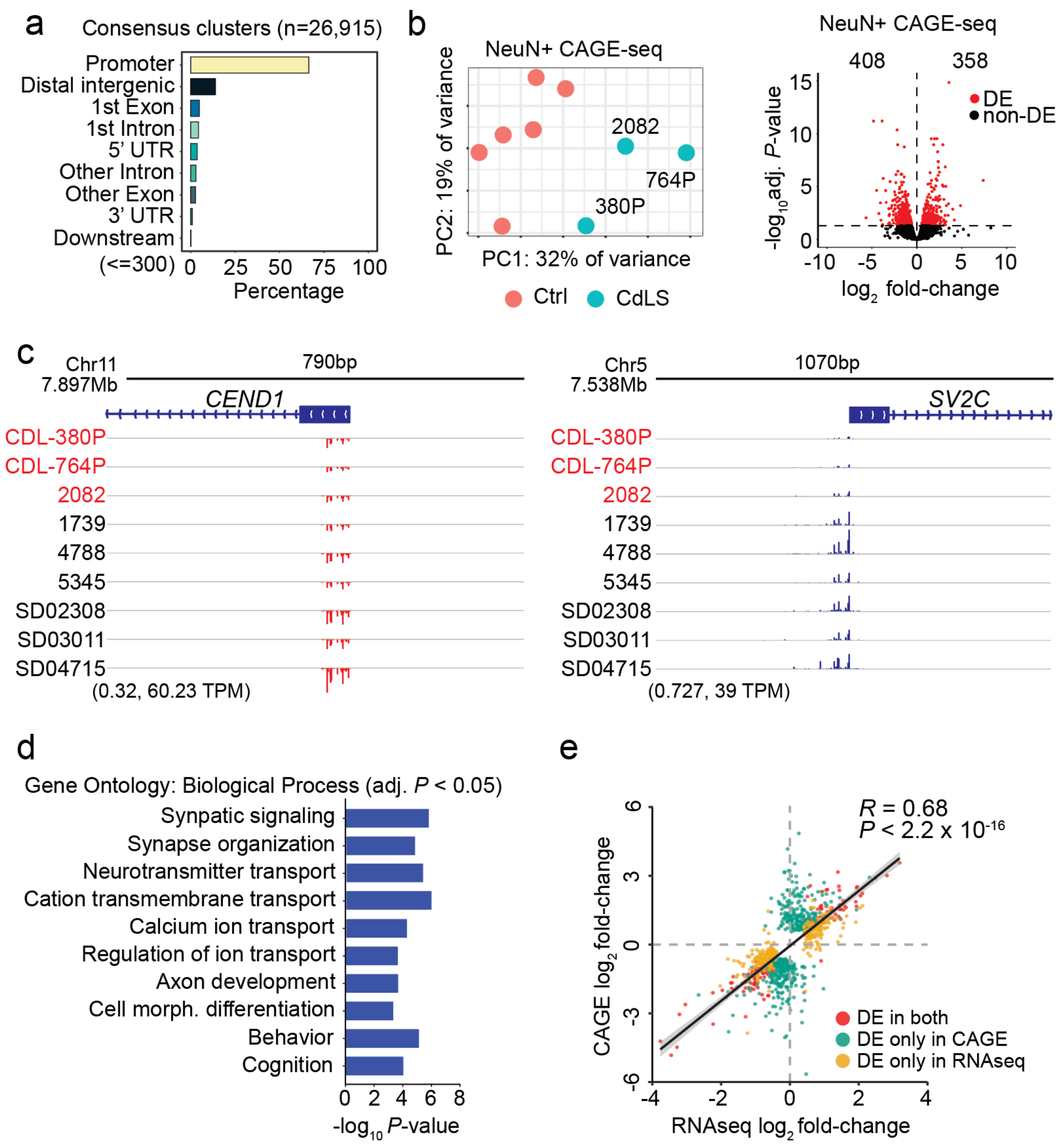

Supplementary Figure 2. CAGE reveals deregulated gene expression in CdLS neurons

a) Genomic annotation of consensus clusters called by CAGEr (ref. 1). A total of 26,915 consensus clusters were annotated with ChIPseeker [2] and the percentage of consensus clusters associated with genomic features was quantified. Consensus clusters associated with database-annotated TSSs or 5' UTRs (n = 18.867) were kept for downstream analysis.

b) Principal component analysis of the 3 CdLS and 6 control samples successfully analysed by CAGE-seq (left) and volcano plot (right) of $\log_2$ fold-change versus adjusted *P*-value obtained from CAGE analysis of 3 patient and 6 control NeuN-positive samples. 408 down- and 358 upregulated genes were identified (adj. P-value < 0.05, Wald Test, Benjamini-Hochberg adjusted). Red indicates differential expression (DE).

c) Screenshots from the Integrative Genomics Viewer (IGV) showing CAGE-defined TSSs (CTSSs) and their corresponding normalized signal (in TPM) across 3 patient (top) and 6 control (bottom) samples. Left: normalized CAGE signal within the *CEND1* promoter region. *CEND1* (cell cycle exit and neuronal differentiation protein 1) is not differentially expressed by DESeq2. Right: normalized CAGE signal within the *SV2C* promoter region. *SV2C* (synaptic vesicle glycoprotein 2C) is significantly downregulated by DESeq2.

d) GO terms represented in the top 25 most enriched biological processes for downregulated CAGE genes identified with clusterProfiler (ref. 3) using 11.634 genes active in control samples as background (adj. *P*-value < 0.05, *q*-value < 0.05, hypergeometric test, Benjamini-Hochberg adjusted).

e) Scatter plot of $\log_2$ fold-change values for genes significantly deregulated in CAGE (green), RNA-seq (orange), or both (red). *R* = *Pearson's* correlation coefficient. *R* and *P*-value were calculated using the two-sided "cor.test" function in R. The linear regression trendline is shown in black and confidence intervals in grey. *P* <2.2e-16.

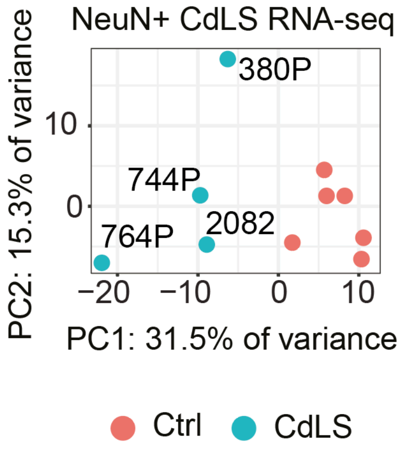

Supplementary Figure 3. Analysis of NeuN-positive RNA-seq data related to Fig. 1.

Principal component analysis of the 4 CdLS and 6 control samples of NeuN-positive nuclei successfully analysed by RNA-seq.

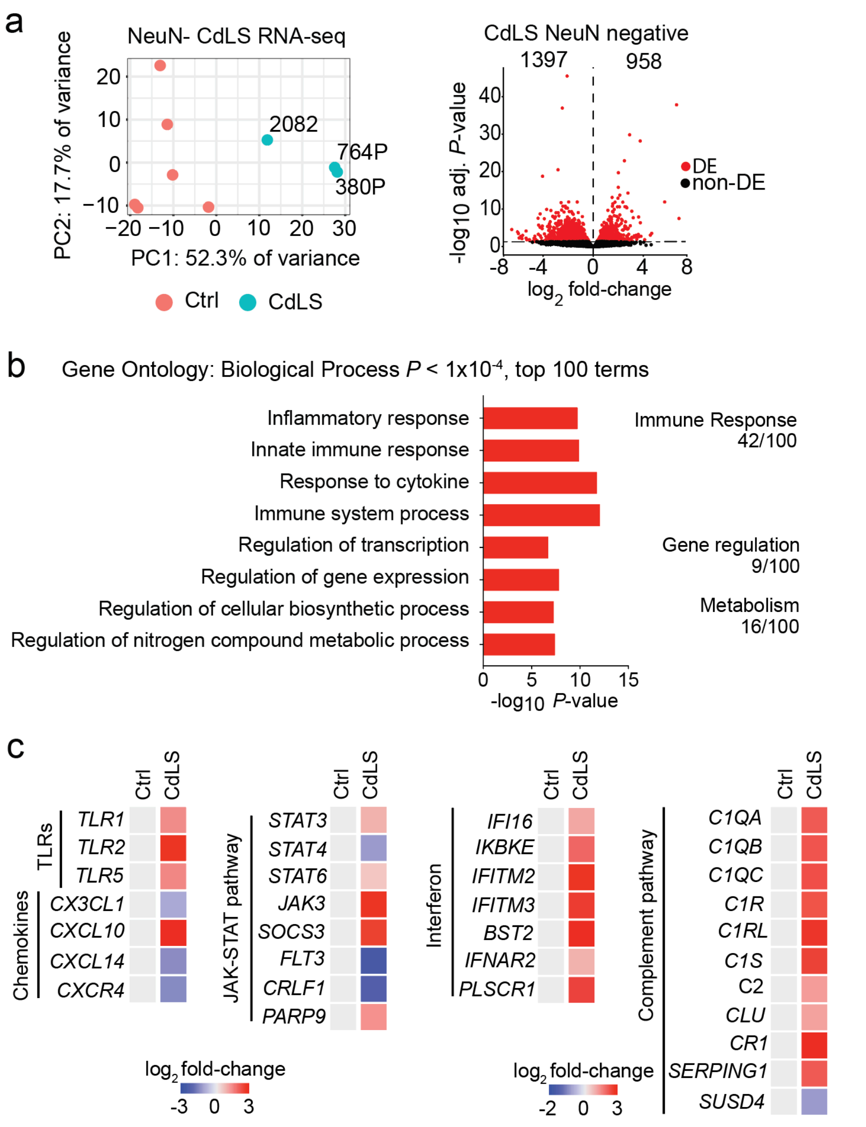

Supplementary Figure 4. RNA-seq of NeuN-negative nuclei from CdLS cortex

a) Principal component analysis of the 3 CdLS and 6 control samples of NeuN-negative nuclei successfully analysed by RNA-seq (left) and volcano plot of gene expression fold-change versus adjusted *P*-value of up- and downregulated genes (right). 1397 genes were down- and 958 genes were upregulated (RUVg k=2, adj. $P < 0.05$, Wald test, Benjamini-Hochberg adjusted). Differentially expressed (DE) genes are shown in red.

b) Bar graph of individual GO terms within broad categories. Terms represented by upregulated genes are shown in red.

c) Heatmap of $\log_2$ fold-changes for representative deregulated genes in NeuN-negative CdLS samples.

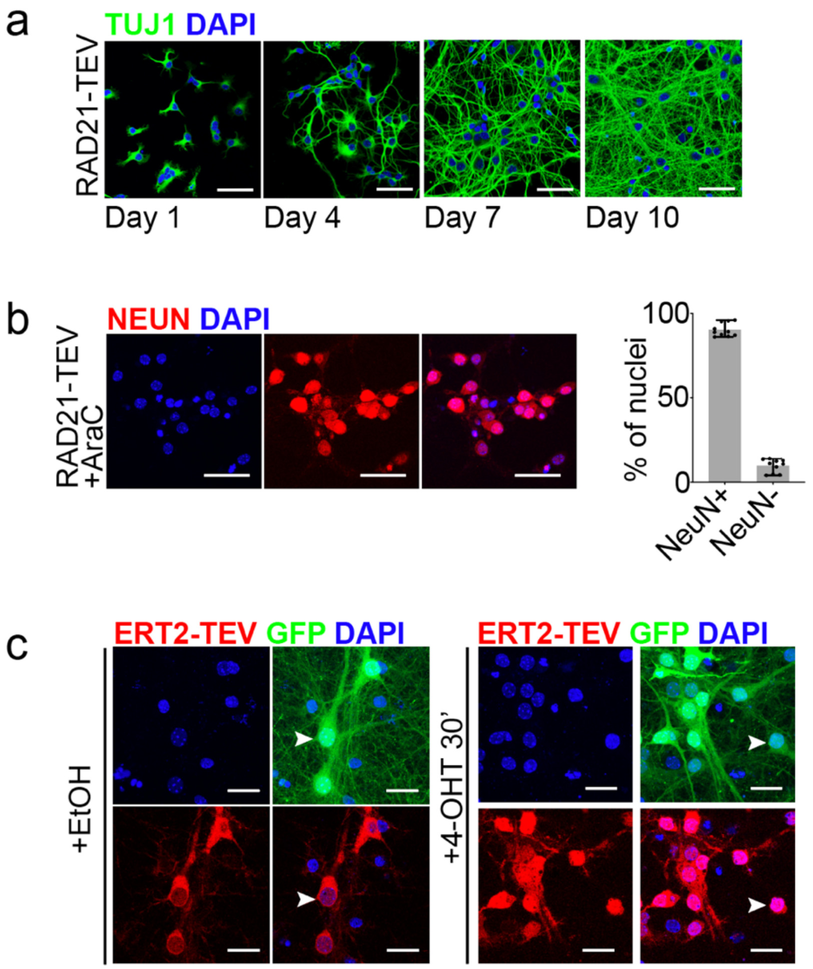

Supplementary Figure 5. Validation of RAD21-TEV neuronal culture and ERt2-TEV translocation

a) Immunofluorescence staining for TUJ1 and DAPI in explant RAD21-TEV neurons showing neuronal maturation over the course of ten days. Representative of 2 independent experiments. Scale bar = 50μm.

b) Immunofluorescence staining for NeuN and DAPI in ten-day old explant RAD21-TEV neuron cultures treated with AraC at day 5. Bar graph plots mean % of NeuN+ and NeuN- nuclei in ten-day old explant RAD21-TEV neuron cultures treated with AraC at day 5. (n =10, mean ± range). Scale bar = 50μm.

c) Immunofluorescence staining for ERt2-TEV and DAPI, with GFP expressed from ERt2-TEV lentiviral transduction. Left: ERT2-TEV is retained in the cytoplasm with vehicle treatment. Right: ERt2-TEV translocates to the nucleus following 4-OHT exposure. White arrow heads indicate example cells. Representative of 2 independent experiments. Scale bar = 25μm.

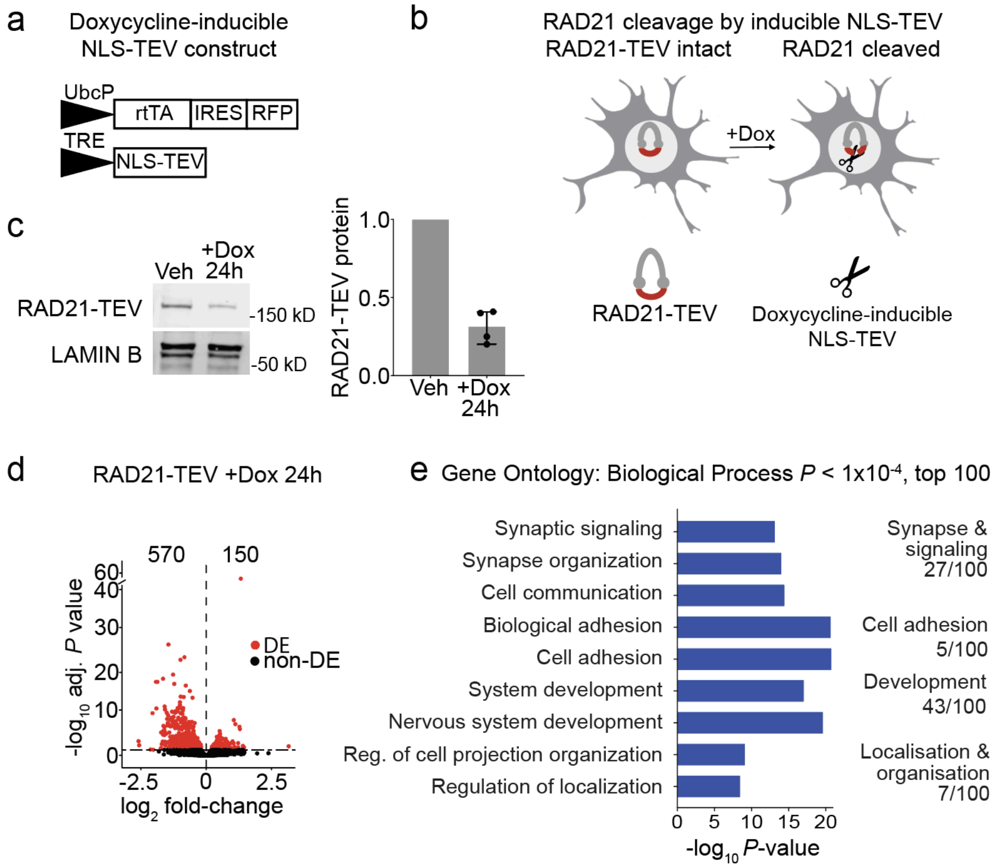

Supplementary Figure 6. Acute RAD21 depletion by NLS-TEV triggers changes in neuronal gene expression

a) Schematic of lentiviral construct for doxycycline inducible NLS-TEV. Tet-On advanced transactivator (rtTA) and RFP are driven by an ubiquitin promoter. NLS-TEV expression is controlled by tet response element (TRE) upon addition of doxycycline.

b) Schematic of doxycycline dependent RAD21-TEV degradation by NLS-TEV.

c) Western blot of RAD21-TEV protein 24h after 6h Dox pulse (100ng/ml). Bar plot of RAD21-TEV protein expression normalised to LAMIN B 24 hours after Dox exposure, ~30% RAD21-TEV protein remained. The mean and expression range (error bars) are shown for n = 4 control and n = 4 NLS-TEV-induced samples.

d) Volcano plot of $\log_2$ fold-change versus adjusted *P* value in RAD21-TEV 24h after 6h Dox pulse (100ng/ml, n = 3). 570 genes were down- and 150 genes were upregulated (adj $P < 0.05$, Wald test, Benjamini-Hochberg adjusted). Differentially expressed (DE) genes are shown in red.

e) Top individual GO terms and broad categories for downregulated genes (blue). There was no significant enrichment for upregulated genes.

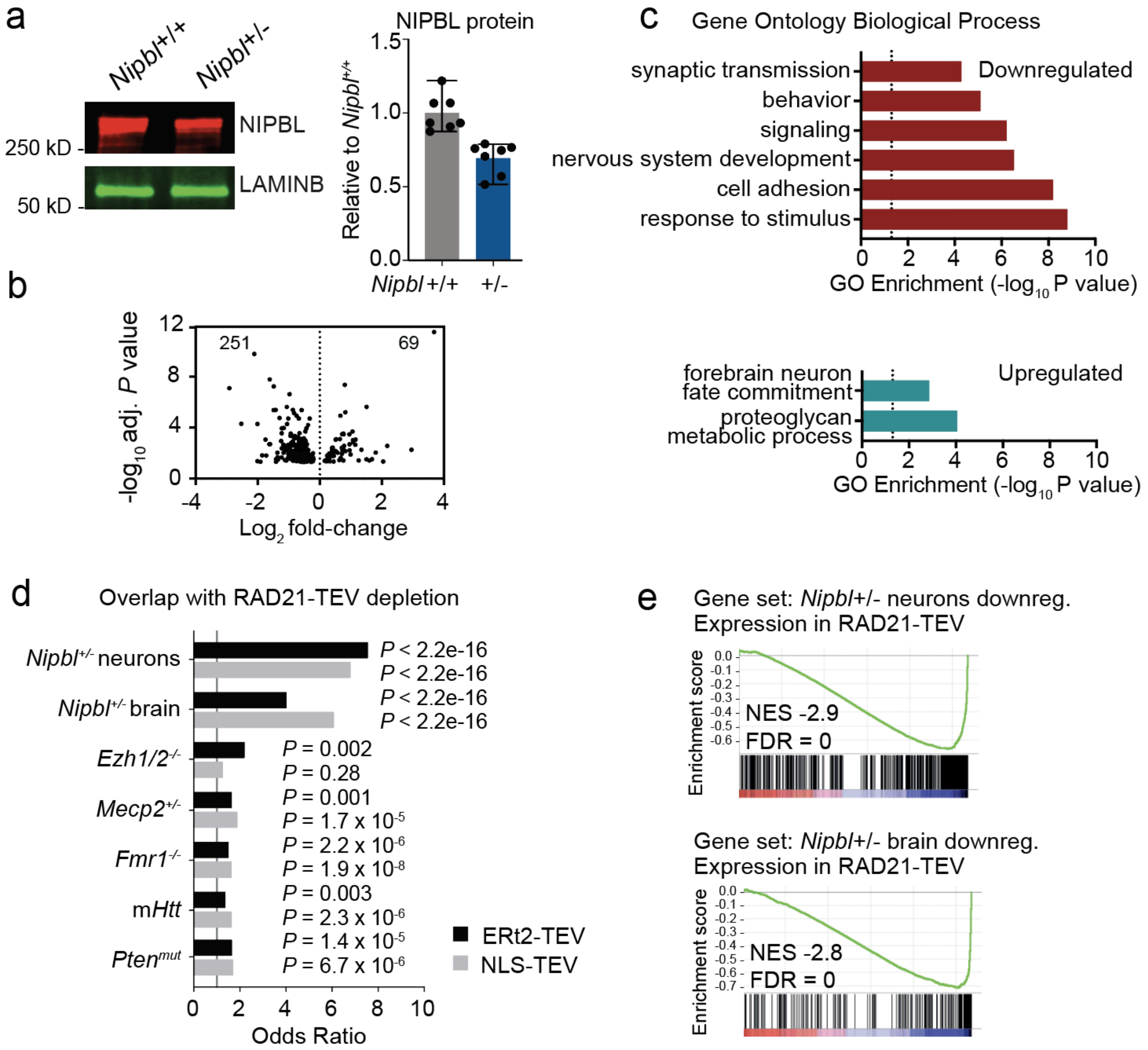

Supplementary Figure 7. Neuronal gene deregulation in acute RAD21-TEV depletion, *Nipbl* heterozygosity and other mouse models of neuronal dysfunction

a) Representative fluorescent western blot for NIPBL and LAMINB proteins (left). NIPBL protein levels quantified by fluorescent western blot analysis and represented as relative to *Nipbl*$^{+/+}$. LAMINB protein levels were used for normalization (right, mean ± range of n = 7 biological replicates).

b) Volcano plot of gene expression fold-change versus adjusted P value of up- and downregulated genes in *Nipbl*$^{+/-}$ neuronal explant cultures analysed by RNA-seq after 10 days in vitro (n = 3 biological replicates). At adj $P < 0.05$, 69 genes were up- and 251 genes were downregulated in *Nipbl*$^{+/-}$ versus *Nipbl*$^{+/+}$ neurons.

c) Gene ontology of biological functions for downregulated and upregulated genes in *Nipbl*$^{+/-}$ neurons

d) Bar graph of overlap between deregulated genes in RAD21-TEV neurons and animal models of neuronal dysfunction including *Nipbl*$^{+/-}$ neurons (this study) *Nipbl*$^{+/-}$ brain [4], deletion of PRC2 components *Ezh1* and *Ezh2* (*Ezh1-2*$^{-/-}$) [5], *Mecp2*$^{+/-}$ (ref. 6), *Fmr1*$^{-/-}$ (ref. 7), mutant *Htt* (m*Htt*), a mouse model of Huntington's disease[8] and mutant *Pten*$^{m3m4/m3m4}$ (*Pten*^{mut}, ref. 9). *P* values were determined by one sided Fisher's exact test.

e) GSEA showing the expression of in RAD21-TEV neurons of genes downregulated *Nipbl*$^{+/-}$ neurons (DEseq2, adj. $P < 0.05$) and in embryonic *Nipbl*$^{+/-}$ brain [4] (DEseq2, adj. $P < 0.05$).

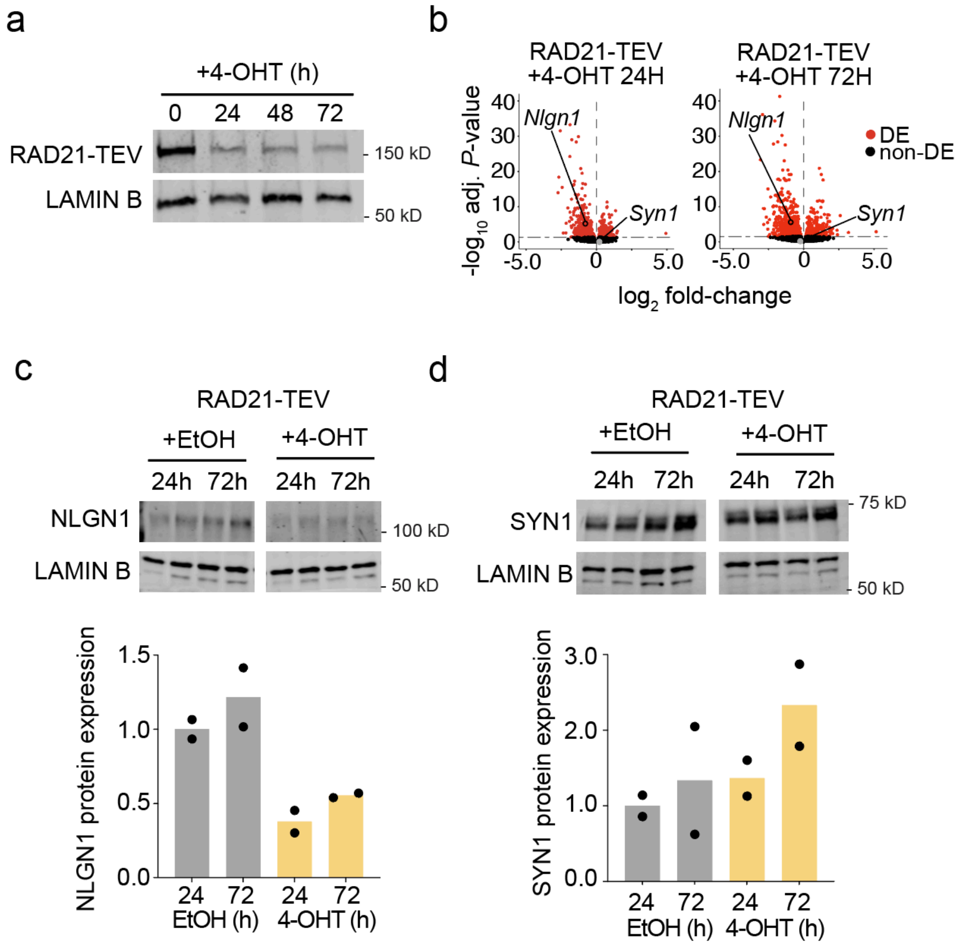

Supplementary Figure 8. RAD21-TEV depletion results alters the expression of protein as well as mRNA.

a) Western blot of RAD21-TEV protein expression at 24, 48 and 72 h of 4-OHT treatment.

b) Volcano plot of gene expression $\log_2$ fold-change versus adjusted *P* value (Wald test, Benjamini-Hochberg adjusted) in RAD21-TEV neurons transduced with ERt2-TEV and treated with 4-OHT for 24h (n = 3, Supplementary Data 8) or 72h (n = 3, Supplementary Data 12). *Nlgn1* is deregulated (adj. $P = 6.42 \times 10^{-6}$, $\log_2$ fold-change = -0.69 at 24h and adj. $P = 0.42 \times 10^{-6}$, $\log_2$ fold-change = -0.69 at 72h) and is highlighted with a black circle, *Syn1* is not deregulated (adj. $P = 0.92$, $\log_2$ fold-change = 0.06 at 24h and adj. $P = 0.79$, $\log_2$ fold-change = -0.1 at 72h) and is highlighted in grey.

c) Western blot of deregulated NLGN1 protein expression 24 and 72 h after 4-OHT or EtOH treatment. Bar plot of NLGN1 protein expression normalised to LAMIN B (n = 2).

d) Western blot of non-deregulated control protein SYN1 following 24 and 72 h after 4-OHT or EtOH treatment. Bar plot of SYN1 protein expression normalised to LAMIN B (n = 2).

Overlap between genes deregulated in RAD21-TEV and human disease

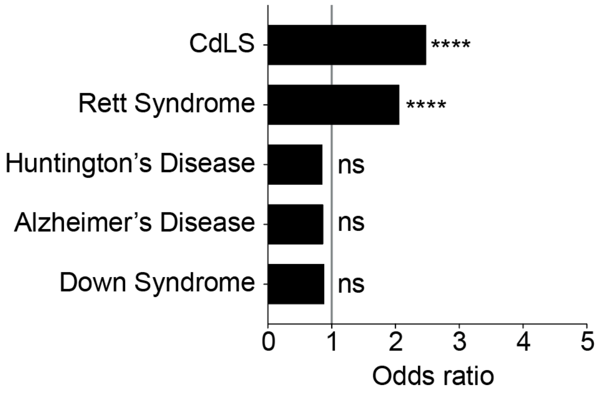

Supplementary Figure 9. Comparison of gene deregulation in acute RAD21-TEV depletion and in human disease

Overlap of deregulated genes between RAD21-TEV neurons (NLS-TEV and ERt2-TEV combined) and neuronal or whole cortical tissue RNAseq of human neurological diseases including NeuN-positive CdLS (P = 4.87e-11), Rett Syndrome (P = 4.64e-10, ref. 6), Huntington’s disease (P = 0.98, ref. 10), Alzheimer’s disease (P = 0.96, ref. 11), and Down Syndrome (P = 0.81, ref. 12). * P <0.05, ** P <0.01, *** P <0.001, **** P <0.0001, one-sided Fisher’s exact test.

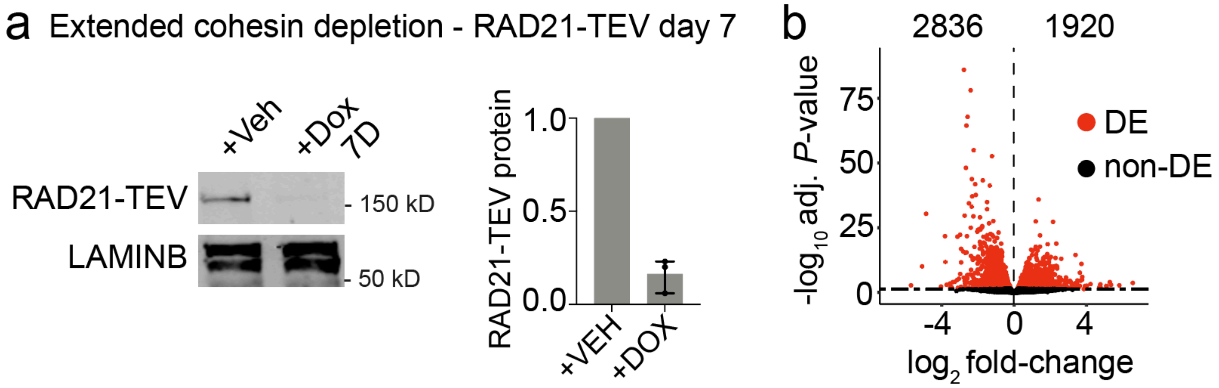

Supplementary Figure 10. Neuronal gene expression does not accommodate to depletion of RAD21.

a) Western blot of RAD21-TEV protein expression 7 days hours after Dox pulse (24h, 1μg/ml). Bar plot of RAD21-TEV protein expression normalised to LAMIN B 7 days after Dox exposure, ~20% RAD21-TEV protein remained (n = 3, d = days, mean ± range).

b) Volcano plot of gene expression $\log_2$ fold-change versus adjusted *P* value in RAD21-TEV +Dox (7 days hours after 24-hour 1μg/ml pulse, n = 3). 2836 genes were down- and 1920 genes were upregulated (adj $P < 0.05$, shown in red, DE = differentially expressed, Supplementary Data 14).

HGNC.symbol	CdLS NeuN+		Function	SFARI	ASD	dup15q	mgi_symbo	ERt2-TEV 24h		NLS-TEV 24h		
	Log2 F-C	adj. P		score				Log2 F-C	adj. P	Log2 F-C	adj. P	Rescue
FAT1	-0.793803253	0.03892864	Cell adhesion	**3**			Fat1	-0.861924	0.031811	-1.054324	2.75E-07	Yes
SOX5	-0.666733736	0.0362642	Transcription	**1**			Sox5	-0.221992	NS	-0.294212	0.017574	Yes
ST8SIA2	-1.748319059	0.00333419	Sphingolipid	**2**			St8sia2	-0.091601	NS	-0.648057	0.000187	Yes
CECR2	1.155825	0.00762567	Transcription	**3**			Cecr2	-1.17458	0.038677	0.169346	NS	NA
PRKD1	-0.589258342	0.01899673	Signaling	**Syndromic**			Prkd1	-0.752496	0.023188	-0.672089	NS	NA
DPP4	-1.537046	3.959E-08	Metabolism	**3**			Dpp4	1.159328	0.042117	1.464866	NS	NA
CELF6	0.470591233	0.04750129	Transcription	**3**			Celf6	0.505136	0.007356	0.20792	NS	NA
ALDH1A3	-2.029866	0.00037112	Metabolism	**Syndromic**			Aldh1a3	-1.100862	0.001143	0.249099	NS	NA
OXTR	0.7886356	0.0113909	Synapse/NT	**2**			Oxtr	-0.869665	0.033963	-0.535398	NS	NA
GABRG3	-0.494385573	0.03394635	Synapse/NT	**2**	**Down**	**Down**	Gabrg3	-0.970309	0.009604	-0.855991	0.004568	Yes
PCDHB10	-0.686989595	0.0198948	Cell adhesion		**Down**	**Down**	Pcdhb18	-0.967581	3.73E-06	-0.924017	4.31E-09	Yes
ATP1B1	-0.589296778	0.01919687	Ion channel		**Down**	**Down**	Atp1b1	-0.443388	0.016885	-0.4911	1.15E-07	Yes
NXPH3	-0.708323763	0.02787885	Signaling		**Down**		Nxph3	-0.888888	7.91E-08	-0.775085	0.001187	Yes
FZD1	0.92282493	0.03361549	Signaling		**Up**		Fzd1	0.899702	6.6E-06	0.693683	0.001588	Yes
PHLDA1	-0.594627864	0.03557529	Signaling		**Up**		Phlda1	-1.177564	0.000312	-1.102917	0.019492	Yes
LGI2	-0.918602029	0.00352707	Synapse/NT			**Down**	Lgi2	-0.502996	NS	-0.654281	0.013963	Yes
PMEPA1	-0.718423149	0.01936601	Signaling			**Down**	Pmepa1	-0.145595	NS	-0.808156	0.02341	Yes
KCNA1	-0.863060667	0.03068165	Ion channel		**Down**	**Down**	Kcna1	-0.091914	NS	-0.515653	0.03293	Yes
CLMP	-1.230116433	0.00209746	Cell adhesion			**Down**	9030425E11	0.088852	NS	-0.312781	0.039734	Yes
NETO2	-0.566935427	0.0049107	Synapse/NT			**Down**	Neto2	-1.090384	7.64E-13	-0.905182	1.83E-07	Yes
CAMK2G	-0.486240671	0.01919687	Signaling		**Down**		Camk2g	0.224785	0.001077	0.196945	NS	NA
GABRA1	-0.513264459	0.03017643	Synapse/NT		**Down**	**Down**	Gabra1	-0.327455	0.042984	-0.184755	NS	NA
ADRA1A	-0.562376885	0.04494848	Synapse/NT		**Up**		Adra1a	-1.450125	NS	-1.602203	1.74E-07	Yes
HTR1A	1.081492501	0.02465718	Synapse/NT				Htr1a	-1.61225	2.09E-09	-1.19949	0.015133	Yes
ETV5	-1.191861948	0.00082508	Transcription				Etv5	-1.111096	4.39E-06	-1.242118	0.00305	Yes
SSTR1	0.580914703	0.04172542	Signaling				Sstr1	-1.355418	2.16E-10	-1.459295	1.96E-10	Yes
SLC35F1	-0.862729774	1.9873E-07	Ion channel				Slc35f1	-0.392999	0.016989	-0.666453	2E-06	Yes
DPY19L1	-1.057156027	5.7684E-07	Signaling				Dpy19l1	-0.976524	3.82E-10	-0.591421	2.03E-05	Yes
ETV1	-1.693428198	6.8596E-09	Transcription				Etv1	-0.963278	1.1E-08	-1.125627	3.88E-05	Yes
NEURL1B	0.722896405	0.02718218	Signaling				Neurl1b	-0.321939	0.046631	-0.442078	8.41E-05	Yes
CTGF	-1.911796384	0.03992878	Signaling				Ctgf	-0.799693	0.009581	-1.047401	0.000197	Yes
KIT	-1.365846558	0.03834312	Signaling				Kit	-0.677588	0.034664	-0.87865	0.000382	Yes
LMO4	-0.624449619	0.04330221	Signaling				Lmo4	-0.778294	0.000308	-0.706432	0.000744	Yes
C8orf34	-0.589094756	0.03898896	Signaling				A830018L1	-0.535352	0.041121	-0.698307	0.02	Yes
LGR5	-1.02789849	0.04253295	Signaling				Lgr5	-1.441919	0.0004	-1.166323	0.020893	Yes
CALB1	1.199029821	1.5637E-05	Signaling				Calb1	-2.17297	0.002732	-1.70523	0.02401	Yes
PALMD	2.106730898	3.3288E-05	Signaling				Palmd	-0.632518	0.00083	-1.129606	0.032338	Yes
OSBPL3	-0.848094524	0.00097849	Signaling				Osbpl3	-0.920222	0.000371	-0.967636	1.14E-08	Yes
KCNG1	0.905077269	0.04517848	Ion channel				Kcng1	-1.029893	2.18E-10	-1.448388	2.7E-08	Yes
NETO1	-0.668723692	0.00155331	Synapse/NT				Neto1	-0.897954	1.87E-05	-0.618843	4.69E-08	Yes
B3GALT2	-0.755423304	0.02002327	Sphingolipid				B3galt2	-1.753183	1.01E-29	-1.234179	8.88E-08	Yes
TSPAN33	0.920382584	0.00168092	Signaling				Tspan33	0.474667	0.01397	0.538281	0.032412	Yes
PEG3	-0.493736249	0.0362642	Transcription				Peg3	0.336426	NS	0.518666	1.42E-06	Yes
CDH24	-1.289075272	0.0017004	Cell adhesion				Cdh24	-0.155702	NS	0.528743	3E-05	Yes
COL12A1	-0.761437665	0.00291979	Cell adhesion				Col12a1	-0.198791	NS	-0.994396	0.002264	Yes
OTOF	1.071584569	0.03530152	Synapse/NT				Otof	0.332461	NS	0.723334	0.0129	Yes
CHRM2	-0.826903865	0.01242275	Synapse/NT				Chrm2	-0.424616	NS	-1.101754	0.000776	Yes
VWA5B2	-0.777185883	2.8447E-06	Signaling				Vwa5b2	0.39167	NS	0.424836	0.001375	Yes
USP43	0.550780133	0.02645709	Signaling				Usp43	-0.57023	NS	-0.851646	0.001509	Yes
TGFBR1	-0.770700784	0.02367098	Signaling				Tgfbr1	0.082952	NS	-0.695604	0.003996	Yes
CHST1	0.573560245	0.01403931	Signaling				Chst1	-0.308097	NS	-0.359626	0.010139	Yes
SNED1	1.094326353	0.00407292	Signaling				Sned1	-0.399297	NS	1.228349	0.011428	Yes
DNA2	0.59305851	0.00697445	Chromatin				Dna2	0.049325	NS	0.86897	0.0116	Yes
FAM105A	0.594107995	0.02169672	Signaling				Fam105a	0.194492	NS	0.629556	0.014062	Yes
EPHA6	0.579168483	0.02465718	Signaling				Epha6	-0.596678	NS	-0.65983	0.014231	Yes
KCNK2	-0.825930728	0.03555103	Ion channel				Kcnk2	-0.312918	NS	-0.421067	0.016502	Yes
AIFM3	-0.82522845	0.00610841	Signaling				Aifm3	0.091698	NS	0.889431	0.02	Yes
LYPD6	-0.936055243	0.01548744	Signaling				Lypd6	-0.256624	NS	-0.86653	0.021205	Yes
RND2	-0.819870951	0.0240464	Signaling				Rnd2	0.249036	NS	0.457882	0.025224	Yes
PTPRE	-0.527609992	0.01413769	Signaling				Ptpre	-0.355473	NS	-0.38858	0.039782	Yes
EPHA7	-0.702651429	0.00479452	Signaling				Epha7	-0.257154	NS	-0.289665	0.041656	Yes
UBASH3B	-1.16341826	0.00104824	Signaling				Ubash3b	-0.085006	NS	-0.3114	0.047351	Yes
ESYT3	0.670757563	0.03892057	Sphingolipid				Esyt3	0.524444	NS	1.399612	0.028887	Yes
PRKAA1	-0.716844285	0.00071262	Sphingolipid				Prkaa1	0.235632	NS	0.393214	0.036976	Yes
ABCG1	0.664943956	0.01550688	Transport				Abcg1	0.205691	NS	0.356729	0.026917	Yes
YPEL1	1.057521	1.8438E-06	Signaling				Ypel1	0.416782	9E-07	0.12401	NS	NA
ROBO3	-1.371404	3.5061E-06	Signaling				Robo3	-1.787881	3.02E-05	-1.297048	NS	NA
COX19	0.570866952	0.04914966	Transport				Cox19	0.186645	0.041035	0.245431	NS	NA
RAMP3	-1.521782472	0.01263868	Signaling				Ramp3	-1.449225	3.26E-06	-1.020448	NS	NA
FAM198B	-1.069614447	1.4525E-05	Signaling				Fam198b	-1.217258	6.52E-05	-0.853865	NS	NA
EGFR	-0.712001753	0.04426687	Signaling				Egfr	-0.955445	0.045796	-0.808042	NS	NA
MYOF	-1.821911303	0.00111126	Signaling				Myof	-1.423443	0.002532	-0.555805	NS	NA
TMEM167A	-0.567766779	0.02423555	Transport				Tmem167	-0.215045	0.005127	-0.262261	NS	NA
HSPA1A	1.481091065	0.0229144	Signaling				Hspa1a	0.644788	0.027885	0.560679	NS	NA
HSPA1B	1.56506796	0.01167575	Signaling				Hspa1a	0.644788	0.027885	0.560679	NS	NA
SV2C	-2.240962532	1.4525E-05	Synapse/NT				Sv2c	-1.143616	0.024083	0.323522	NS	NA
PIK3R3	-0.871026446	0.0059316	Signaling				Pik3r3	0.540831	0.048375	0.27183	NS	NA
RPH3A	-0.6299956	0.03210071	Signaling				Rph3a	-0.656549	0.00434	-0.663562	NS	NA
SHF	0.8859735	0.00929482	Signaling				Shf	0.326513	0.039829	0.214538	NS	NA
ZNF184	0.5963439	0.02257451	Transcription				Zfp184	0.37547	0.005656	0.191612	NS	NA
HCRTR1	-2.230947	9.0256E-05	Synapse/NT				Hcrtr1	-0.599401	0.017323	0.202374	NS	NA

Supplementary Table 1. Features of genes that are deregulated in CdLS and in response to acute cohesin depletion in mouse neurons.

The table shows human and mouse gene symbols, $\log_2$ fold-change (F-C) in CdLS, ERt2-Tev- and NLS-TEV-mediated RAD21-TEV cleavage at 24h, adjusted P-value (adj P), SFARI score (ref. 13), deregulation in idiopathic ASD (ASD, ref. 14) and syndromic ASD with duplication of chromosome 15q.11.2-13.1 (syndromic, ref. 14)**.** NS = not significant**.** NA = not applicable (rescue could not be assessed because the gene was not deregulated 24h after NLS-TEV-induced RAD21-TEV cleavage).

Supplementary References

1 Haberle, V., Forrest, A., Hayashizaki, Y., Carninci, P. & Lenhard, B. CAGEr: precise TSS data retrieval and high-resolution promoterome mining for integrative analyses. *Nucleic Acids Research* 43, e51-e51 (2015).

2 Balwierz, P. et al. Methods for analyzing deep sequencing expression data: constructing the human and mouse promoterome with deepCAGE data. *Genome Biology* 10, R79 (2009).

3 Yu, G., Wang, L., Han, Y. & He, Q. clusterProfiler: an R Package for Comparing Biological Themes Among Gene Clusters. *OMICS: A Journal of Integrative Biology* 16, 284-287 (2012).

4 Kawauchi, S. et al. Multiple Organ System Defects and Transcriptional Dysregulation in the Nipbl+/- Mouse, a Model of Cornelia de Lange Syndrome. *PLoS Genetics* 5, e1000650 (2009).

5 von Schimmelmann, M. et al. Polycomb repressive complex 2 (PRC2) silences genes responsible for neurodegeneration. *Nature Neuroscience* 19, 1321-1330 (2016).

6 Renthal, W. et al. Characterization of human mosaic Rett syndrome brain tissue by single-nucleus RNA sequencing. *Nature Neuroscience* 21, 1670-1679 (2018).

7 Korb, E. et al. Excess Translation of Epigenetic Regulators Contributes to Fragile X Syndrome and Is Alleviated by Brd4 Inhibition. *Cell* 170, 1209-1223.e20 (2017

8 Langfelder, P. et al. Integrated genomics and proteomics define huntingtin CAG length–dependent networks in mice. *Nature Neuroscience* 19, 623-633 (2016).

9 Tilot, A. et al. Neural transcriptome of constitutional Pten dysfunction in mice and its relevance to human idiopathic autism spectrum disorder. *Molecular Psychiatry* 21, 118-125 (2015).

10 Labadorf, A. et al. RNA Sequence Analysis of Human Huntington Disease Brain Reveals an Extensive Increase in Inflammatory and Developmental Gene Expression. *PLOS ONE* 10, e0143563 (2015).

11 Mathys, H. et al. Single-cell transcriptomic analysis of Alzheimer’s disease. *Nature* 570, 332-337 (2019).

12 Olmos-Serrano, J. et al. Down Syndrome Developmental Brain Transcriptome Reveals Defective Oligodendrocyte Differentiation and Myelination. *Neuron* 89, 1208-1222 (2016).

13 Banerjee-Basu, S. & Packer, A. SFARI Gene: an evolving database for the autism research community. *Disease Models & Mechanisms* 3, 133-135 (2010).

14 Parikshak, N. et al. Genome-wide changes in lncRNA, splicing, and regional gene expression patterns in autism. *Nature* 540, 423-427 (2016).
