# Supplementary material for: Neuronal genes deregulated in Cornelia de Lange Syndrome respond to removal and re-expression of cohesin
Source: Nat Commun. 2021 May 18;12:2919. doi: 10.1038/s41467-021-23141-9 (PMC8131595; doi:10.1038/s41467-021-23141-9)
Supplement: Supplementary file 3 — Reporting Summary [file 41467_2021_23141_MOESM3_ESM.pdf]

nature research

Corresponding author(s): Matthias Merkenschlager

Last updated by author(s): 2021/27/2

Reporting Summary

Nature Research wishes to improve the reproducibility of the work that we publish. This form provides structure for consistency and transparency in reporting. For further information on Nature Research policies, see our Editorial Policies and the Editorial Policy Checklist.

Statistics

For all statistical analyses, confirm that the following items are present in the figure legend, table legend, main text, or Methods section.

n/a	Confirmed	
☐	☒	The exact sample size (*n*) for each experimental group/condition, given as a discrete number and unit of measurement
☐	☒	A statement on whether measurements were taken from distinct samples or whether the same sample was measured repeatedly
☐	☒	The statistical test(s) used AND whether they are one- or two-sided<br>*Only common tests should be described solely by name; describe more complex techniques in the Methods section.*
☒	☐	A description of all covariates tested
☐	☒	A description of any assumptions or corrections, such as tests of normality and adjustment for multiple comparisons
☐	☒	A full description of the statistical parameters including central tendency (e.g. means) or other basic estimates (e.g. regression coefficient) AND variation (e.g. standard deviation) or associated estimates of uncertainty (e.g. confidence intervals)
☐	☒	For null hypothesis testing, the test statistic (e.g. F, t, r) with confidence intervals, effect sizes, degrees of freedom and P value noted<br>*Give P values as exact values whenever suitable.*
☒	☐	For Bayesian analysis, information on the choice of priors and Markov chain Monte Carlo settings
☒	☐	For hierarchical and complex designs, identification of the appropriate level for tests and full reporting of outcomes
☐	☒	Estimates of effect sizes (e.g. Cohen's *d*, Pearson's *r*), indicating how they were calculated

Our web collection on statistics for biologists contains articles on many of the points above.

Software and code

Policy information about availability of computer code

Data collection	Leica Application Suite X (LAS X, v2.7) software was used for confocal image acquisition. Image Studio™ Software (v5.2) for Western blot image acquisition, quantification and analysis.
Data analysis	Prism 8<br>Microsoft Excel v16.16<br>FIJI v1.O<br>lmageStudiolite vS.2.5<br>CellProfiler v2.2<br>FlowJo v10.6<br>BD FACSDiva8.0<br>Sequencher vS.2.4<br>Mutation Taster2<br>CAGE:<br>Bowtie2 v 2.3.5.1<br>Samtools v 1.10<br>liftOver<br>R package - CAGEr v 1.20<br>R package - ChIPseeker v 1.22.1<br>R package - DESeq2 v 1.26.0<br>R package - limma v 3.42.0<br>Rpackage - clusterProfiler v 3.14.3<br>RNASeq:<br>Tophat2 version 2.0.11<br>Samtools version 1.2

GSEA version 2.2.0
R version 3.6.2
Bioconductor version 3.9
R package - Rsubread version 1.24.2
R package - DESeq2 version 1.24.0
R package - GOseq vl.24
R package - RUVseq version 1.18.0
R package - biomaRt version 2.40.5
R package - GeneOverlap version 1.20.0
R package - ComplexHeatmap version 2.0.0
ATACseq:
trim_galore version 0.4.4
Samtools version 1.2
bowtie2 version 2.2.9
Picard version 1.90
MACS2 2.0.10
deeptools 3.2.1
R version 3.6.2
Bioconductor version 3.9
R package ATACseqQC version 1.8.5
R package - Rsubread version 1.24.2
R package - ChIPseeker version 1.20.0
4C:
4Cseqpipesoftware suite v0.7

For manuscripts utilizing custom algorithms or software that are central to the research but not yet described in published literature, software must be made available to editors and reviewers. We strongly encourage code deposition in a community repository (e.g. GitHub). See the Nature Research guidelines for submitting code & software for further information.

Data

Policy information about availability of data

All manuscripts must include a data availability statement. This statement should provide the following information, where applicable:

- Accession codes, unique identifiers, or web links for publicly available datasets
- A list of figures that have associated raw data
- A description of any restrictions on data availability

RNA-seq, ATAC-seq and 4C-seq data have been deposited at GEO under accession number GSE150130. CAGE data has been deposited at ArrayExpress under accession number E-MTAB-9045.

Field-specific reporting

Please select the one below that is the best fit for your research. If you are not sure, read the appropriate sections before making your selection.

☒ Life sciences ☐ Behavioural & social sciences ☐ Ecological, evolutionary & environmental sciences

For a reference copy of the document with all sections, see nature.com/documents/nr-reporting-summary-flat.pdf

Life sciences study design

All studies must disclose on these points even when the disclosure is negative.

Sample size	No statistical test was used to determine sample size. Three or more biological replicates per group (as indicated in the figure legends) were used to provide sufficient data to allow statistical inference.
Data exclusions	CAGE: Standard filtering of sequencing data was performed with bowtie2, which involved retaining only uniquely mapped reads (using default parameters and allowing zero mismatches per 22 nucleotide seed sequence). SLIC-CAGE data was first filtered to exclude lowly expressed CTSSs. This was done by discarding CTSSs with < 0.1 TPM in 1 sample (threshold = 0.1, thresholdIsTpm = TRUE, nrPassThreshold = 1, keepSingletonsAbove = 1 within clusterCTSS function of CAGEr). The above criteria were less stringent than the default thresholds used in CAGEr, which was due to the observation that many genes detected as differentially-expressed in the RNAseq data remained undetected by SLIC-CAGE when using default CAGEr parameters. Low-fidelity tag clusters with normalized signal < 5 TPM were discarded when constructing consensus clusters (according to the standard CAGEr workflow), but were allowed to contribute to the total signal of a consensus cluster (excludeSignalBelowThreshold=FALSE within aggregateTagClusters function of CAGEr). Sample CDL-744P was excluded from the analysis due to its low correlation with the rest of the samples on the CTSS level and an apparent prevalence of tag clusters with low interquantile widths. This suggested an enrichment with artificially sharp promoters caused by a lower library complexity for this sample, i.e. not all TSSs within a tag cluster are captured due to loss of material during library preparation (Cvetesic et al 2018, Genome Res).Prior to differential expression analysis on the consensus cluster (promoter) level, consensus clusters were filtered according to three criteria: 1. Since we were interested in differential expression of gene promoters only, consensus clusters that were not associated with promoters or 5'UTRs (according to ChIPseeker annotation) were removed. 2. Consensus clusters located on chromosomes X and Y were discarded in order to alleviate differences in expression caused by the individual's gender. 3. Consensus clusters associated with glial genes were discarded, in order to alleviate differences in expression caused by contamination from glial cells. In order to perform the functional annotation of CdLS-deregulated genes within the context of neuronal cells, we used genes active in control samples (> 2 TPM) as a background set for the GO enrichment analysis. RNAseq: No data were excluded except for standard quality control filtering during sequencing analysis. This includes filtering genes

	with low read counts due to their low statistical power using a DESeq2 independent filtering approach. Standard filtering also involves removing reads aligning to multiple positions.
Replication	Number of replicates and independent experiments detailed in figure legends, a minimum of three biological replicates were used for each experiment.
Randomization	No randomization was required. Mice used for primary neuron culture were chosen according to genotype, human samples used were chosen according to disease status.
Blinding	Investigators were not blinded to group allocation. We considered that blinding was not relevant to this study because we used quantitative assays for all experiments and the computational framework was identical for all samples and replicates

Reporting for specific materials, systems and methods

We require information from authors about some types of materials, experimental systems and methods used in many studies. Here, indicate whether each material, system or method listed is relevant to your study. If you are not sure if a list item applies to your research, read the appropriate section before selecting a response.

Materials & experimental systems

n/a	Involved in the study	
☐	☒	Antibodies
☐	☒	Eukaryotic cell lines
☒	☐	Palaeontology and archaeology
☐	☒	Animals and other organisms
☐	☒	Human research participants
☒	☐	Clinical data
☒	☐	Dual use research of concern

Methods

n/a	Involved in the study	
☒	☐	ChIP-seq
☐	☒	Flow cytometry
☒	☐	MRI-based neuroimaging

Antibodies

Antibodies used	Immunoblot: Primary antibodies were LAMIN B (1:5000, Santa Cruz Biotechnology, sc-6216), LAMIN B (1:5000, Santa Cruz Biotechnology, sc-374015) and c-MYC (1:500, Santa Cruz Biotechnology, sc-40), rabbit polyclonal to NIPBL (1:1000; A301-779A, Bethyl Laboratories), mouse monoclonal to NLGNl (1:100, Santa Cruz biotechnology sc-365110) and goat polyclonal antibody to SYN1(l:2500, Synaptic Systems, 106103). Secondary antibodies were donkey anti-goat lgG (H+L) Alexa Fluor 680 (1:10,000, Thermofisher, A-21804), goat anti-mouse lgG (H+L) Alexa Fluor 680 (1:10,000 Thermofisher, A-28183) and donkey-anti mouse IgG (H +L) Alexa Fluor 790 (1:10,000 Thermofisher A-11371). Nuclei sorting: NEUN (1:200, Abeam, ab190195). lmmunocytochemistry: Primary antibodies were TUJl (1:500, Biolegend, 801213), V5 (1:250, Sigma-Aldrich, V8012) and NEUN (1:1000, Abeam, ab104224). Secondary antibodies were goat anti-mouse lgG (H+L) Alexa Fluor 488 (ThermoFisher, A-1101) and goat anti-mouse lgG (H+L) Alexa Fluor 568 (ThermoFisher, A-11004).
Validation	All antibodies are commercially available and were used according to the manufacturers' recommendations and had previously been tested in mouse. All companies provided quality certificates for the antibodies used. Optimal dilutions for each antibody were used and are stated in the methods section.

Eukaryotic cell lines

Policy information about cell lines

Cell line source(s)	HEK293T cells were kindly provided by Prof. Jesus Gil (London Institute of Medical Sciences, MRC)
Authentication	HEK293T were not authenticated
Mycoplasma contamination	HEK293T cells tested negative for mycoplasma
Commonly misidentified lines (See ICLAC register)	No commonly misidentified cell lines were used

Animals and other organisms

Policy information about studies involving animals; ARRIVE guidelines recommended for reporting animal research

Laboratory animals	Laboratory bred mice of the appropriate genotype were maintained under SPF conditions and 12h light/dark cycle. Embryos (Rad21TEV: E14.5; Nipbl+/-: E17.5-18.5) were used to derive cells and tissues.
Wild animals	The study did not involve wild animals.
Field-collected samples	The study did not involve samples collected from the field.

Ethics oversight | Ethical approval was granted by Home Office, UK, and the Imperial College London Animal Welfare and Ethical Review Body (AWERB).

Note that full information on the approval of the study protocol must also be provided in the manuscript.

Human research participants

Policy information about studies involving human research participants

Population characteristics

Frontal cortex samples were collected from the following 10 patients:
5345: Female, 48 years old, healthy
4788: Female, 48 years old, healthy
1739: Female 48 years old, healthy
2082: Female, 48 years old, Cornelia de Lange Syndrome
SD047/15: Male, 19 years old, healthy
SD023/08: Female, 28 years old, healthy
SD030/11: Male, 30 years old, healthy
CDL-380P: Male, 19 years old, Cornelia de Lange Syndrome
CDL-744P: Female, 24 years old, Cornelia de Lange Syndrome
CDL-764P: Female, 31 years old, Cornelia de Lange Syndrome

Recruitment

CDL-380P, CDL-744P and CDL-764P were obtain from Prof. Ian Krantz, Children's Hospital of Philadelphia. 5345, 4788, 1739 and 2082 were obtained from the NIH NeuroBioBank. SD047/15, SD023/08 and SD030/11 were obtained from Edinburgh Brain Bank. Healthy controls were selected to age match Cornelia de Lange Syndrome patients.

Ethics oversight

Anonymised human tissue was used in accordance with the Human Tissue Act (UK) and with approval by the Imperial College London Research Ethics Committee.

Note that full information on the approval of the study protocol must also be provided in the manuscript.

Flow Cytometry

Plots

Confirm that:

☒ The axis labels state the marker and fluorochrome used (e.g. CD4-FITC).

☒ The axis scales are clearly visible. Include numbers along axes only for bottom left plot of group (a 'group' is an analysis of identical markers).

☒ All plots are contour plots with outliers or pseudocolor plots.

☒ A numerical value for number of cells or percentage (with statistics) is provided.

Methodology

Sample preparation

Isolation of nuclei: 50-250mg of pre-frontal cortical grey matter was homogenised in homogenisation buffer (250mM sucrose, 25mM KCl, 5mM MgCl2, l0mM Tris buffer pH 8.0, lµM DTT, lX Proteinase Inhibitor w/o EDTA (Roche), 0.4U µ1-1 RNaseln (ThermoFisher) 0.2U µl-1 Superasin (ThermoFisher), lµM DAPI) and centrifuged through an iodixanol gradient (Sigma). Pelleted nuclei were washed and then stained with NeuN antibody (Abeam, ab190195) in staining buffer (PBS, 1% BSA, 0.2U/µl RNaseln (Thermofisher), NeuN antibody (1:200) for one hour at 4oC. For negative controls antibody was excluded. Nuclei were then sorted on BD Fusion, and collected in wash buffer (PBS, 1%BSA, 0.2U/µl RNaseln (Thermofisher) before RNA extraction.

Instrument

FACSAria Fusion

Software

BD FACSDiva8.0
FlowJo v10

Cell population abundance

Nuclei were isolated from homogenized post-mortem human pre-frontal cortical tissue. Nuclei represented ~30-50% of initial sample, of which ~70-90% were single nuclei. Within this population ~30-50% were positive for the marker NeuN, and ~30-50% were NeuN negative.

Gating strategy

Nuclei were first gated for being DAPI positive (DAPI) and then gated to exclude doublets (SSC-A/SSC-H). To enrich for neuronal nuclei, nuclei were stained for neuonal nuclei marker NeuN. This resulted in two clearly defined nuclei populations, with neuronal nuclei were identified as being NeuN positive and gated for sorting as neuronal nuclei, NeuN negative nuclei were gated and sorted.

☒ Tick this box to confirm that a figure exemplifying the gating strategy is provided in the Supplementary Information.
